# Validation of Walk Score^®^ for Estimating Neighborhood Walkability: An Analysis of Four US Metropolitan Areas

**DOI:** 10.3390/ijerph8114160

**Published:** 2011-11-04

**Authors:** Dustin T. Duncan, Jared Aldstadt, John Whalen, Steven J. Melly, Steven L. Gortmaker

**Affiliations:** 1 Department of Society, Human Development, and Health, Harvard School of Public Health, 677 Huntington Avenue, Kresge Building 7th Floor, Boston, MA 02115, USA; E-Mail: sgortmak@hsph.harvard.edu; 2 Harvard Prevention Research Center on Nutrition and Physical Activity, Harvard School of Public Health, 401 Park Drive, Landmark Center, 4th Floor West, Boston, MA 02215, USA; 3 Department of Geography, University at Buffalo, State University of New York, 105 Wilkeson Quad, Buffalo, NY 14261, USA; E-Mails: geojared@buffalo.edu (J.A.); whalenjf@gmail.com (J.W.); 4 Department of Environmental Health, Harvard School of Public Health, 401 Park Drive, Landmark Center, 4th Floor West, Boston, MA 02215, USA; E-Mail: sjmelly@hsph.harvard.edu

**Keywords:** neighborhood walkability, GIS, Walk Score^®^, validity, multi-city

## Abstract

Neighborhood walkability can influence physical activity. We evaluated the validity of Walk Score^®^ for assessing neighborhood walkability based on GIS (objective) indicators of neighborhood walkability with addresses from four US metropolitan areas with several street network buffer distances (*i.e.*, 400-, 800-, and 1,600-meters). Address data come from the YMCA-Harvard After School Food and Fitness Project, an obesity prevention intervention involving children aged 5–11 years and their families participating in YMCA-administered, after-school programs located in four geographically diverse metropolitan areas in the US (n = 733). GIS data were used to measure multiple objective indicators of neighborhood walkability. Walk Scores were also obtained for the participant’s residential addresses. Spearman correlations between Walk Scores and the GIS neighborhood walkability indicators were calculated as well as Spearman correlations accounting for spatial autocorrelation. There were many significant moderate correlations between Walk Scores and the GIS neighborhood walkability indicators such as density of retail destinations and intersection density (p < 0.05). The magnitude varied by the GIS indicator of neighborhood walkability. Correlations generally became stronger with a larger spatial scale, and there were some geographic differences. Walk Score^®^ is free and publicly available for public health researchers and practitioners. Results from our study suggest that Walk Score^®^ is a valid measure of estimating certain aspects of neighborhood walkability, particularly at the 1600-meter buffer. As such, our study confirms and extends the generalizability of previous findings demonstrating that Walk Score is a valid measure of estimating neighborhood walkability in multiple geographic locations and at multiple spatial scales.

## 1. Introduction

Physical activity is associated with numerous well-documented health benefits [1–3]. However, most Americans do not meet national physical activity guidelines [4]. An important goal of public health, therefore, is to promote physical activity.

A large amount of research shows that environmental features of neighborhoods can influence physical activity among children, adolescents and adults [5–12]. Collectively, these features that promote various forms of physical activity (such as walking) can be referred to as ‘neighborhood walkability’ and often include access to walking destinations such as retail stores and parks, and community design features such as street connectivity and sidewalk access [13]. Metrics used to assess neighborhood walkability vary considerably, including use of self-reported information and use of systematic field observation, also known as environmental audits [14]. Self-reported measures of neighborhood walkability can be implicated in same-source bias [15], and there are other noteworthy problems common in survey research, including issues with reliability, validity, low response rates and a biased sample of respondents [16]. Systematic field observations are well-known to be very laborious (*i.e.*, time-intensive and have multiple logistical constraints), often can require significant specialized training and are notorious for being very costly. Geographic information systems (GIS) data, increasingly, is used to evaluate neighborhood walkability [17]. While GIS data can be useful for measuring neighborhood walkability, a caveat is that using GIS also requires specialized expertise and also can be time-intensive [17]. In addition, GIS data layers might not be readily accessible for certain geographic regions and can be expensive to acquire.

Walk Score has become increasingly recognized in the study of walkability due to its accessibility, international scale and use of dynamic (or up-to-date) data that is constantly being corrected. This popular tool allows a user to enter any query location into the online interface on the Walk Score publicly available website (www.walkscore.com) and receive the Walk Score assigned to that location, free of charge. The Walk Score algorithm, which produces a score of 0 to 100, calculates a score of walkability based on distance to various categories of amenities (e.g., schools, stores, parks and libraries) that are weighted equally and summed. To date, there is a very limited amount of research that has examined the validity of Walk Score to measure aspects of neighborhood walkablity [18,19]. These studies are useful and indicate that Walk Score can validly measure walkable amenities such as retail stores [19] and several community design features such as street connectivity [18]. However, to our knowledge, the existing published research examining the validity of Walk Score were conducted in a single location (*i.e.*, Providence, RI, US). It is possible that the validity of Walk Score might vary by geographic locations, limiting the generalizablity of the previous research. Additionally, previous research has evaluated Walk Score compared to GIS-derived walkablity indicators based on a 1-mile buffer only [18,19]. However, neighborhoods are defined variously in neighborhood health effects research and Walk Score might not be a useful assessment of neighborhood walkability for certain populations. To illustrate, smaller spatial scales (e.g., 400- and 800-meters) may be particularly salient to children and the elderly who may have limited neighborhood mobility and thus the finite Walk Score algorithm (which is calibrated to a 1-mile neighborhood definition) might not be relevant to these populations. This all highlights the importance of understanding the validity of Walk Score at multiple spatial scales.

The purpose of this study was to evaluate the validity of Walk Score for assessing neighborhood walkability based on several GIS (objective) indicators of neighborhood walkability for a relatively large number of addresses from four US metropolitan areas. We conducted our analysis overall and for each of the metropolitan areas with several street network buffer distances (*i.e.*, 400-, 800-, and 1600-meter buffers).

## 2. Methods

### 2.1. Address Data

This study used address data collected as part of the YMCA-Harvard After School Food and Fitness Project, a multi-site, quasi-experimental, after-school obesity prevention intervention targeting children aged 5–11 years and their families [20]. The intervention focused on changing after-school environments to promote physical activity and healthy eating as well as to reduce television and computer time and to foster social connectedness. It was delivered to after-school programs, administered by the YMCA and located in four geographically diverse metropolitan areas in the US. The four metropolitan areas consisted of YMCAs that self-selected to participate in the intervention; there were 32 program sites across the metropolitan areas. The metropolitan areas varied in size and in degree of urbanicity (program sites were both in urban and suburban areas). For anonymity, we discuss the metropolitan areas by general geographic region only: the Pacific Northwest (n = 180), the Midwest (n = 170), the South (n = 238) and the East (n = 166). Thus, there were 754 full baseline addresses collected from parents of children in the after-school sites. Baseline data were collected in the fall of 2006, and the follow-up was in the spring of 2007. The analyses for the present study include full baseline addresses collected from the parents of children in the after-school sites who have geocodable addresses (n = 733), not just those who actually participated in various intervention activities. Although the sample is families of children who participated in the YMCA after-school programs, the geocoded families’ residential addresses represent neighborhoods across the metropolitan areas (the participants live in urban, suburban and even some rural areas).

### 2.2. Address Geocoding

We geocoded the participants with full baseline street address information (*i.e.*, street address, city, state, zone improvement plan [ZIP] code) collected from the parents of children in the after-school sites. Our geocoding methods have been described in detail elsewhere [21]. All addresses were preprocessed before geocoding by cleaning them to improve their quality. First, we removed any address that had PO boxes. We then reviewed the data for misspelled address information using Google Maps and remedied incorrect home addresses (e.g., incorrect street names). In addition, we removed all extraneous geographic characteristics (e.g., apartment numbers) and standardized the spelling to the United States Postal Service format (e.g., we changed ‘Street’ to ‘St’, ‘Avenue’ to ‘Ave’, and ‘Circle’ to ‘Cir’). Addresses were then geocoded to the street level and assigned longitude and latitude coordinates, using the Tele Atlas US street address locator via the ArcGIS Online World Geocoding service with ArcGIS version 9.3 (Environmental Systems Research Institute, Redlands, CA, USA). Addresses were matched automatically using a minimum match score of 65, spelling sensitivity of 60 and side offset of 10 feet—the default settings of ArcGIS. We then manually restricted addresses in this analysis to those with a match score ≥80. For all addresses with candidate ties that had match scores ≥80 (n = 9), we performed interactive rematching in ArcGIS, which resulted in one address change. In the final step, we used Google Earth Pro to geocode the addresses with match scores below 80 and those that ArcGIS were unable to geocode. This study includes 700 addresses geocoded by ArcGIS and 33 by Google Earth Pro.

### 2.3. Neighborhood Walkability Assessment using Geographic Information Systems

Objective neighborhood walkability indicators were created via geographic information systems (GIS) using ArcGIS 9.3. GIS data were analyzed using the North American Datum (NAD) 1983 state plane coordinate system for each of the four metropolitan areas. This study includes total retail walking destinations (e.g., clothing stores, pharmacy/drug stores, bookstores) per square kilometer, total service walking destinations (e.g., post offices, banks, credit unions) per square kilometer, total cultural/educational walking destinations (e.g., movie theaters, schools, libraries) per square kilometer, parks per square kilometer, median pedestrian route directness (median of the ratio of distance between one point and another via the street network and straight-line distance between the two points; values closer to 1.00 represent a more direct route or a more connected network), intersection density (the number of street intersections per square kilometer; intersections are defined as street network nodes with three or more associated street segments excluding highways), count of cul de sacs (based on nodes associated with only one street segment), average speed limit (miles per hour), highway density (percentage of area that is highway traveled right of way; class 1 and 2 highways were used), residential density (US census block group occupied housing units per square kilometer were weighted proportionally for the child’s defined neighborhood) and population density (US census block group total population per square kilometer were weighted proportionally for the child’s defined neighborhood). We limited the retail, service and cultural/educational walking destinations to locations with fewer than 250 employees to filter out large businesses (e.g., Costco, Home Depot) as business with greater than 250 employees can take away from the walkability of a neighborhood (e.g., by having large parking lots) [22]. Retail, service and cultural/educational walking destinations data come from ESRI Business Analysis InfoUSA Business Locations 2006. ESRI Data and Maps information, from ESRI, has spatial datasets representing several built environment features. InfoUSA (http://infousa.com) is a company that provides listings of private and public businesses (verified yearly by telephone), with 6-digit NAICS codes as well as numbers of employees. Locations of these businesses had been geocoded and were available as a spatial dataset through the ESRI Business Analyst Extension. Data on parks, intersection density, cul de sacs, average speed limit, and highway density come from ESRI Data and Maps 2006; median pedestrian route directness data are derived from ESRI Business Analyst Info USA Business Locations 2006. Residential density and population density data as previously described come from 2000 US Census. This geospatial dataset includes GIS-derived walkability indicators for neighborhoods defined as 400-, 800- and 1600-meter street network buffers. We specifically selected 400- and 800-meters for our small spatial scales because these distances are considered a proximal neighborhood environment for children and adolescents [23,24], among other populations such as older adults [25–28]. The 1600-meter buffer was used because it is approximately 1-mile, which is consistent with the Walk Score algorithm. The street network buffers were created from StreetMap streets excluding highways and ramps using the ArcGIS Network Analyst Extension. The street network buffers consisted of 50-meter buffers around street center lines that extend along the network 400-, 800- and 1600-meters from the geocoded home addresses.

### 2.4. Neighborhood Walkability Assessment using Walk Score

Walk Score^®^ (www.walkscore.com) is a publicly available large-scale method for calculating walkability. Walk Score was developed by Front Seat Management (www.frontseat.org), a software development company based in Seattle, WA, which focuses on software with civic applications. Walk Score uses publicly available data to assign a score to a location based on the distance to and variety of nearby commercial and public frequently-visited facilities. Data sources used by Walk Score include Google, Education.com, Open Street Map and Localeze. Facilities are divided into five categories: educational (e.g., schools), retail (e.g., grocery, drug, convenience and bookstores), food (e.g., restaurants), recreational (e.g., parks and gyms) and entertainment (e.g., movie theaters). The Walk Score algorithm then calculates the distance to the closest of each of the five facilities, using straight-line distances, and calculates a linear combination of these distances weighted both by facility type priority and a distance decay function [29]. The result is normalized to fit a 0 to 100 scale, with 0 being the lowest (lowest walkability/car dependent) and 100 being the highest (most walkable). If one of each of the five facilities is within a quarter-mile radius from the input location, that location receives a perfect 100 score. If no facilities are within a one-mile radius of the input location, that location will be assigned a score of zero. The location can be entered as geographic coordinates, or as an address which is then geolocated using Google Geolocation [30]. Front Seat provides an application programming interface (API), which can be used to query the Walk Score database through URL calls, eliminating the need to use the website interface [31]. In order to use the Walk Score API, the user must first obtain a key number, which can be requested on the Walk Score website. This unique key is used in all API calls, and has a limit on the number of uses per 24 hours. Using a scripting language, the user is able to paste a set of geographic coordinates along with the key number into an API call to quickly retrieve a Walk Score for each location. For this study, a program was created within the R programming language (R Foundation for Statistical Computing, Vienna, Austria), which queries the Walk Score database for each address used in the study. The script then scans the API response, which is in the form of an HTML page, and extracts the corresponding Walk Score. We obtained the walkability scores from Walk Score in mid May of 2011 using the Walk Score API and the geographic coordinates.

### 2.5. Statistical Analysis

First, we conducted descriptive statistics (e.g., means, standard deviations, ranges) for the neighborhood walkability metrics assessed via GIS and Walk Score. We then computed non-parametric Spearman product correlations between the GIS neighborhood walkability indicators and the Walk Score values because the neighborhood walkability data had a nonnormal distribution. We recognize the features of the built environments often cluster, indicating the presence of spatial autocorrelation. The presence of spatial autocorrelation violates the assumption of independent observations which can impact the findings (e.g., the presence of spatial autocorrelation can result in inflated degrees of freedom in the conventional correlation tests of the significance which can lead to overestimation of significance of effects) [32,33]. We evaluated the presence of spatial autocorrelation via the Global Moran’s *I* statistic, with a k nearest neighbor of four spatial weights matrix and 999 Monte Carlo simulations were used to compute a pseudo p-value [34,35]. The Clifford and Richardson adjustment method was used to account for spatial autocorrelation, with six spatial lags used in generating correlation matrices and also the four nearest neighbor weights matrix [32]. In this methodology, the sample size is adjusted to account for the spatial dependence between observations. The corresponding t-statistics and p-values will change based on the adjusted sample size. All analyses were conducted for the data overall and for each of the four metropolitan areas, and for our three different neighborhood definitions. We report both r_S_-values and significance values. A significance level of 0.05 was selected for all analyses. Descriptive statistics and a-spatial statistical analyses were conducted in SAS version 9.2 (SAS Institute Inc., Cary, NC, USA). Spatial analyses were conducted using the R statistical program version 2.13 with the *spdep* package [36].

## 3. Results

### 3.1. Descriptive Statistics of Walk Score and GIS Neighborhood Walkability Indicators

Descriptive statistics of the Walk Score and GIS neighborhood walkability indicators are presented in Table 1 and Table 2, respectively. The mean Walk Score for the overall data was 38.84 (SD = 23.81). There was a large range in Walk Scores for the overall data with a minimum Walk Score of 0 and a maximum of 97. GIS neighborhood walkability indicators and Walk Scores varied by metropolitan area. The metropolitan area in the East had a mean Walk Score of 53.01, while 26.19 was the Walk Score mean for the metropolitan area in the Midwest.

### 3.2. Correlation between Walk Scores and GIS Neighborhood Walkability Indicators

Table 3 shows the conventional Spearman correlation between Walk Scores and GIS neighborhood walkability indicators as well as their p-values, including all data and for each geographic region at various spatial scales. For the overall data (n = 733 addresses) for the 400-meter buffer, significant correlations were found for total retail walking destinations per square kilometer (r_S_ = 0.53, p < 0.0001), total service walking destinations per square kilometer (r_S_ = 0.27, p < 0.0001), total cultural/educational walking destinations per square kilometer (r_S_ = 0.44, p < 0.0001), parks per square kilometer (r_S_ = 0.24, p < 0.0001), median pedestrian route directness (r_S_ = 0.24, p < 0.0001), intersection density (r_S_ = 0.51, p < 0.0001), average speed limit (r_S_ = 0.47, p < 0.0001), highway density (r_S_ = 0.33, p < 0.0001), residential density (r_S_ = 0.65, p < 0.0001) and population density (r_S_ = 0.64, p < 0.0001). No statistically significant correlation was found for count of cul de sacs (r_S_ = 0.01, p = 0.7024).

For the 800-meter buffer for the overall data, significant correlations were found for total retail walking destinations per square kilometer (r_S_ = 0.67, p < 0.0001), total service walking destinations per square kilometer (r_S_ = 0.53, p < 0.0001), total cultural/educational walking destinations per square kilometer (r_S_ = 0.53, p < 0.0001), parks per square kilometer (r_S_ = 0.37, p < 0.0001), intersection density (r_S_ = 0.59, p < 0.0001), count of cul de sacs (r_S_ = 0.14, p = 0.0002), average speed limit (r_S_ = 0.53, p < 0.0001), highway density (r_S_ = 0.39, p < 0.0001), residential density (r_S_ = 0.65, p < 0.0001) and population density (r_S_ = 0.64, p < 0.0001); there was not a statistically significant correlation found for median pedestrian route directness (r_S_ = −0.01, p = 0.7908).

For the overall data using the 1600-meter buffer, significant correlations were found for total retail walking destinations per square kilometer (r_S_ = 0.80, p < 0.0001), total service walking destinations per square kilometer (r_S_ = 0.67, p < 0.0001), total cultural/educational walking destinations per square kilometer (r_S_ = 0.69, p < 0.0001), parks per square kilometer (r_S_ = 0.51, p < 0.0001), intersection density (r_S_ = 0.65, p < 0.0001), count of cul de sacs (r_S_ = 0.37, p < 0.0001), average speed limit (r_S_ = 0.47, p < 0.0001), highway density (r_S_ = 0.43, p < 0.0001), residential density (r_S_ = 0.65, p < 0.0001) and population density (r_S_ = 0.64, p < 0.0001). However, there was no statistically significant correlation for median pedestrian route directness (r_S_ = −0.05, p = 0.2166).

The magnitude of the correlation coefficient, significance level and sometimes even direction varied by geographic region in these unadjusted Spearman correlations. Notably, we found significant global spatial autocorrelation in Walk Scores overall and for each geographic region, as well as significant spatial autocorrelation in most GIS built environment indicators across regions and spatial scales. The Global Moran’s *I* for Walk Score overall was 0.79 (p = 0.001) and for the Pacific Northwest, Midwest, South and East the Global Moran’s *I* was 0.70, 0.82, 0.63 and 0.79, respectively (all p = 0.001). However, the Spearman correlations accounting for spatial autocorrelation produced similar findings; the p-values, although more conservative, did not change the overall findings for most GIS indicators (data not shown). Generally then, these analyses demonstrate many significant and moderate correlations between Walk Score and the GIS neighborhood walkability indicators, although the magnitude varied by the GIS indicator of neighborhood walkability. Correlations generally became stronger with a large spatial scale, and there were some geographic differences.

## 4. Discussion

While Walk Score uses novel web-based geospatial technologies to estimate neighborhood walkability and has emerged as a potential tool to be used in public health, very limited empirical evidence exists examining its validity. In order to evaluate the validity of Walk Score, we examined correlations between neighborhood walkability metrics as measured via geographic information systems compared to the Walk Score algorithm. We found that Walk Score was valid in measuring certain aspects of walkability overall, and for each metropolitan area examined in this study. However, it is necessary to highlight that the magnitude of the correlations varied by the GIS indicator of neighborhood walkability and correlations did vary some by geographic region. In this study, there was a major difference in population densities in the four metropolitan areas studied, and it appears that the correlations were higher in the high population density regions. While Walk Score was valid at each of the neighborhood definitions (*i.e.*, 400-, 800-, and 1600-meter street network buffers) for certain aspects of neighborhood walkability, there were higher correlations at the 1600-street network buffer. This is not surprising, as the Walk Score algorithm goes until 1-mile, which is approximately 1600 meters, and this finding suggests that the algorithm works best for the intended neighborhood definition. We were surprised there were some geographic differences in correlations across the four metropolitan areas.

Our study extends previous research because we examined the validity of Walk Score for a geospatial dataset from several metropolitan areas and at multiple neighborhood definitions. Our study compares to the previous research examining the validity of Walk Score in that most correlations were significant. In terms of the magnitude, for the 1600-meter buffer, our correlations were oftentimes lower than the previous research that used a 1-mile buffer [18,19]. One potential reason for this is that the previous research computed Pearson correlations while we computed Spearman correlations. Additionally, in this study we examined some features related to neighborhood walkability not examined in the previous research (e.g., median pedestrian route directness, cul de sac count, average speed limit, and highway density). Although median pedestrian route directness varied by geographic locale and spatial scale, for the data overall it was not significantly related to Walk Scores for the 1600-meter buffer, which highlights the limited utility of Walk Score for measuring overall walkability, as pedestrian route directness is an important aspect of walkabiltiy. Moreover, the correlations between Walk Score and cul de sac count overall were moderate and significant at the 1600-meter buffer level, which further underscores that Walk Score is not a useful proxy for overall neighborhood walkability. We also found significant moderate correlations between Walk Scores and average speed limit as well as Walk Scores and highway density overall, which may also hinder one’s ability to walk in their neighborhood. Therefore, our findings indicate that Walk Score is a useful proxy for only certain neighborhood walkability indicators (e.g., retail destinations, intersection density, residential density). Of note, the prior research, to our knowledge, that has examined the validity of Walk Score for neighborhood walkability manually retrieved the Walk Scores. There is potential for research assistant keystroke error when manually obtaining the Walk Scores. A novel aspect of our study is that we retrieved Walk Scores via the Walk Score API, which can overcome the key stroke error limitation, because it eliminates the need to enter locations one at a time into the website’s interface. Also noteworthy is that obtaining Walk Scores via the Walk Score API was quick and therefore is cost-effective. We believe that the Walk Score API is a tremendous tool for retrieving mass Walk Score data. Another novel and important difference between our study and the existing published studies evaluating the validity of Walk Score is that we examined and accounted for spatial autocorrelation. Although the findings for the spatial autocorrelation adjustment were near identical as the conventional correlation approach, we believe that computing correlations adjusted for spatial autocorrelation is a major strength of the present research because most studies evaluating correlations in geospatial data do not examine and if necessary account for potential spatial autocorrelation in the data.

Although walkability is a complex construct that does not have an agreed upon definition [13], it is important to have metrics that capture features related to walking because walking is the most frequently adopted type of regular physical activity [37] and because some research suggests that pedestrian-oriented communities support walking [5–12]. Our findings are useful to public health researchers and practitioners because recent research demonstrates that composite walkability metrics are more predictive of walking behaviors than single walkability metrics [38] and because Walk Score is free, quick and easy to use. Public health researchers, practitioners and policymakers, regardless of their level of technical experience in geospatial technologies, can easily utilize the Walk Score website. For example, researchers can easily assess the walkability of a person’s neighborhood. Practitioners and policymakers can identify and intervene in areas with limited neighborhood resources. The website can also be used by community groups for community resource assessment and advocacy, potentially advocating for land use policies supportive of multiple types of destinations for the purpose of increased walkability but also to economically strengthen neighborhoods. Archived Walk Scores over time would be a significant enhancement that can be used for a variety of purposes, including allowing researchers to study changes in built environment on walkability and its associated impacts on physical activity and overall health. In addition, Walk Score can also be integrated in physical activity promotion intervention programs. As noted by previous researchers, for example, Walk Score could be used as an intervention tool to inform participants about their access to nearby amenities related to physical activity [18]. Previous research suggests low correlations between objective and perceived neighborhood features [39–43]. Researchers interested in spatial analysis may be likely to employ spatial sampling techniques [44–46]; Walk Score could be a resource for conducting spatial sampling. We note that this study is subject to limitations.

We recognize that GIS datasets can have errors [47–49]. Data from ESRI and other sources used to measure features of the built environment, including Walk Score, might have errors of omission, features that no longer exist and positional errors. Geospatial datasets need to be used with caution and need to be ground-truthed. However, we have no reason to believe that the nature of any potential misclassification is different among the built environment data used in this study. Spatio-temporal mismatches between the GIS neighborhood walkability indicators and Walk Scores is also a limitation. However, this might not make that much of a difference in practice as the built environment likely changes slowly, especially perhaps in established metropolitan areas. We acknowledge though that changes in the built environment can occur as a result of a recession as well as other circumstances including suburban development and gentrification. The spatial autocorrelation usually inherent in built environments and walkability of neighborhoods is another important consideration. Although we computed correlation coefficients adjusted for spatial autocorrelation, we recognize that there are other approaches to account for spatial autocorrelation in correlation analysis and also that the results of spatial approaches can be influenced by the spatial weights matrix. We used the Clifford and Richardson adjustment method, which has been previously applied [50,51], because it does not require strong assumptions about the form of the spatial autocorrelation; e.g., as would a partial correlation adjusted for the geographic coordinates. K nearest neighbor was chosen as the structure for spatial relationships because: (a) the number of neighbors for each unit is constant with this specification; (b) this specification represents the influence of one’s most immediate neighbors; and (c) this specification results in everyone having neighbors [52]. We specifically used a k nearest neighbor spatial weights matrix specification of four, because it has previously been suggested that a spatial weights matrix specification between four and six neighbors is optimal and because it is accepted that applying an under-specified (fewer neighbors) rather than an over-specified (extra neighbors) weights matrix is better (e.g., for increased power) [53,54].

Walk Score is a composite measure. The complexities of composite measures may reduce the transferability for public health and planning practitioners and policymakers alike, as composite measures can minimize the importance of any single neighborhood walkability measure, such as street connectivity. However, previous research has shown that composite measures of neighborhood walkability are more predictive of walking than a single measure [38]. Additionally, it remains an empirical question whether people equally weight all destinations and if the destinations specified in the Walk Score^®^ algorithm are salient to people, with varied socio-demographic characteristics such as gender and race/ethnicity. However, it is important to note that Walk Score does include a variety of desired destinations. Many of the destinations used in the Walk Score^®^ algorithm have been found to predict walking [55–57]. The Walk Score algorithm does not consider the size *i.e.*, percentage of a given area, of its destinations (such as parks) which may matter for physical activity and overall health; it also does not consider the frequency of use of destinations. As walkability is a complex construct, the Walk Score algorithm misses certain variables likely to influence active pedestrian neighborhood transportation including crime, neighborhood aesthetics, traffic, physical terrain, and natural walking barriers such as highways and bodies of water. Additionally, Walk Score calculations are based on straight-line distances between housing units and various destinations. With a grant from Robert Wood Johnson Foundation’s Active Living Research Program, however, Walk Score has developed a beta version of “Street Smart” Walk Score, which includes features related to pedestrian friendliness. The “Street Smart” Walk Score takes into account walking distances, intersection density, and average block length when calculating Walk Scores. The Street Smart will calculate “network distances” rather than straight-line distances, which would improve the validity of these scores. Further research could address some of the limitations in this study, examining the validity of Walk Score with other geospatial datasets. Confirmation of these findings in other locations will be important to gaining a greater confidence in Walk Scores ability to measure neighborhood walkability, as a the potential for spatial bias in the selection and coverage of a diverse set of areas within the metropolitan areas is a limitation of our study and therefore our findings might not be generalizeable to other locales. Future Walk Score validation research is especially needed using geospatial datasets that vary in degree of urbanicity (including rural areas) and done recognizing the global context (including non-US based studies). It is not clear how transferable past and our findings are to non-US contexts. Importantly, future research can begin to understand the effect of neighborhood walkability derived from Walk Score on health and behavior (e.g., physical activity, obesity) among various populations. To date, no published studies that we are aware has used neighborhood walkability information captured by Walk Score as a predictor of health outcomes. We caution that future studies should choose outcomes that are relevant to the Walk Score^®^ algorithm (such as perhaps utilitarian walking as opposed to walking for exercise) and perhaps used for only certain populations (such as urban adults who might walk up to 1-mile).

## 5. Conclusions

Walk Score, a web-based walkability assessment tool, is free and publicly available for public health researchers and practitioners. Results from our study suggest that Walk Score is a valid measure of estimating certain aspects of neighborhood walkability, particularly at the 1600-meter buffer. As such, our study confirms and extends the generalizability of previous findings demonstrating that Walk Score is a valid measure of estimating neighborhood walkability in multiple geographic locations and at multiple spatial scales.

## Figures and Tables

**Table 1:** Descriptive statistics of Walk Scores, including all data and for each geographic region.

	M (SD)	Range
**Overall (n = 733)**	38.84 (23.81)	0–97
**Pacific Northwest (n = 172)**	45.39 (24.50)	0–97
**Midwest (n = 167)**	26.19 (19.80)	0–74
**South (n = 230)**	33.02 (17.86)	0–91
**East (n = 164)**	53.01 (24.70)	0–91

Abbreviations: SD, Standard Deviation; M, Mean.

**Table 2:** Descriptive statistics of GIS Neighborhood Walkability Indicators, including all data and for each geographic region.

	400-meter Network Buffer		800-meter Network Buffer		1600-meter Network Buffer	
	M (SD)	Range	M (SD)	Range	M (SD)	Range
**Overall (n = 733)**						
Retail destinations (density)	5.08 (12.18)	0–107.84	5.11 (9.35)	0–118.79	5.42 (6.13)	0–55.55
Service destinations (density)	0.89 (4.20)	0–60.27	0.88 (2.53)	0–33.80	1.06 (1.59)	0–16.27
Cultural/educational destinations (density)	3.27 (6.33)	0–50.97	3.73 (4.98)	0–24.63	3.84 (4.11)	0–26.19
Parks (density)	0.97 (2.81)	0–19.97	0.60 (1.24)	0–8.34	0.48 (0.68)	0–3.88
Median pedestrian route directness	1.31 (0.67)	1–12.04	1.39 (0.47)	1–6.95	1.37 (0.31)	1–4.56
Intersection density	60.59 (30.87)	0–200.26	54.83 (24.83)	0–152.69	50.64 (21.91)	6.57–137.16
Cul de sacs (count)	2.85 (2.42)	0–13.00	9.23 (6.51)	0–42.00	34.94 (21.16)	1–111.00
Average speed limit (mph)	26.92 (2.47)	21.67–41.18	27.07 (2.01)	22.27–35.94	27.32 (1.59)	22.86–35.37
Highway density	25.96 (84.01)	0–676.04	31.89 (71.60)	0–621.70	38.38 (60.12)	0–400.15
Residential density	76.95 (67.52)	0.11–373.94	75.84 (63.86)	0.11–343.05	73.02 (58.36)	0.22–382.55
Population density	1,470 (1,438)	1.85–8,346	1,451 (1,377)	1.85–7,172	1,384 (1,229)	5.06–6,020
**Pacific Northwest (n = 172)**						
Retail destinations (density)	5.83 (13.58)	0–73.49	6.07 (13.00)	0–118.79	6.38 (8.71)	0–55.55
Service destinations (density)	1.07 (5.27)	0–60.27	1.00 (3.19)	0–33.80	1.27 (2.23)	0–16.27
Cultural/educational destinations (density)	2.69 (6.14)	0–32.09	3.30 (4.90)	0–24.22	3.52 (4.41)	0–26.19
Parks (density)	2.90 (4.68)	0–19.97	1.68 (1.86)	0–8.34	1.21 (0.87)	0–3.88
Median pedestrian route directness	1.43 (1.27)	1–12.04	1.43 (0.65)	1–6.95	1.41 (0.32)	1–2.83
Intersection density	63.56 (26.74)	0–149.13	58.49 (20.07)	6.51–109.74	52.80 (16.48)	9.47–100.16
Cul de sacs (count)	3.55 (2.64)	0–12.00	11.86 (7.63)	0–42.00	46.40 (24.60)	3.00–111.00
Average speed limit (mph)	27.08 (2.44)	22.60–35.93	27.17 (1.82)	23.41–33.16	26.95 (1.26)	23.72–31.59
Highway density	19.44 (77.91)	0–481.67	20.75 (50.39)	0–328.65	18.18 (28.65)	0–150.37
Residential density	76.63 (60.15)	0.11–319.02	74.59 (57.43)	0.11–343.05	71.67 (56.54)	0.22–382.55
Population density	1,472 (1,056)	1.85–6,055	1,445 (1,020)	1.85–6,670	1,408 (966.17)	5.06–5,850
**Midwest (n = 167)**						
Retail destinations (density)	3.42 (13.77)	0–107.84	3.33 (8.77)	0–57.59	3.80 (4.97)	0–21.44
Service destinations (density)	0.85 (4.33)	0–33.18	0.70 (2.09)	0–11.56	0.79 (1.41)	0–7.61
Cultural/educational destinations (density)	1.77 (3.85)	0–19.80	1.95 (2.46)	0–11.31	2.45 (1.66)	0–8.66
Parks (density)	0.21 (1.20)	0–7.65	0.13 (0.55)	0–3.42	0.08 (0.18)	0–0.90
Median pedestrian route directness	1.32 (0.58)	1–3.78	1.42 (0.57)	1–6.08	1.41 (0.33)	1–2.83
Intersection density	44.39 (15.17)	0–82.00	40.96 (10.26)	0–71.29	38.59 (7.79)	6.57–53.39
Cul de sacs (count)	1.93 (1.55)	0–7.00	6.13 (4.41)	0–23.00	21.75 (10.77)	1–53.00
Average speed limit (mph)	25.70 (1.62)	21.67–32.14	25.79 (1.60)	22.27–33.50	26.33 (1.46)	22.86–33.38
Highway density	5.77 (41.32)	0–366.94	13.63 (54.00)	0–441.36	24.67 (49.45)	0–268.08
Residential density	30.68 (18.77)	1.45–121.37	31.06 (19.03)	1.45–124.38	30.97 (17.54)	1.45–96.77
Population density	577.51 (339.65)	25.16–1,915	581.16 (330.59)	25.16–1,776	571.51 (293.82)	25.03–1,452
**South (n = 230)**						
Retail destinations (density)	3.86 (11.04)	0–63.93	4.04 (7.61)	0–52.32	4.48 (4.58)	0–31.22
Service destinations (density)	1.17 (4.53)	0–37.88	1.01 (2.93)	0–30.52	1.10 (1.40)	0–6.74
Cultural/educational destinations (density)	2.71 (6.30)	0–50.97	3.46 (4.56)	0–21.53	3.31 (2.69)	0–16.46
Parks (density)	0.33 (1.57)	0–13.94	0.24 (0.72)	0–3.86	0.25 (0.38)	0–1.59
Median pedestrian route directness	1.26 (0.25)	1–2.19	1.42 (0.37)	1–3.10	1.36 (0.27)	1–2.49
Intersection density	52.17 (21.62)	0–128.59	46.31 (12.66)	9.38–77.97	42.91 (9.31)	7.55–65.15
Cul de sacs (count)	3.22 (2.64)	0–13.00	10.04 (6.53)	0–32.00	38.41 (21.36)	1–83.00
Average speed limit (mph)	27.11 (2.81)	25.00–41.18	27.25 (1.86)	25.00–35.94	27.52 (1.19)	25.00–33.45
Highway density	19.78 (80.49)	0–676.03	23.03 (69.53)	0–621.70	27.12 (53.97)	0–290.40
Residential density	81.81 (55.92)	2.69–336.25	82.17 (53.67)	2.72–332.16	82.19 (48.45)	3.02–273.75
Population density	1,264 (715.00)	60.47–3185	1,256 (654.16)	61.05–2,658	1,240 (574.41)	65.34–2493
**East (n = 164)**						
Retail destinations (density)	7.68 (9.77)	0–55.63	7.42 (6.69)	0–35.33	7.39 (5.08)	0–18.39
Service destinations (density)	0.37 (1.33)	0–9.02	0.77 (1.25)	0–5.97	1.08 (1.11)	0–5.87
Cultural/educational destinations (density)	6.20 (7.59)	0–27.69	6.37 (6.34)	0–24.63	6.35 (5.79)	0–22.32
Parks (density)	0.62 (1.50)	0–6.17	0.47 (0.73)	0–3.45	0.43 (0.49)	0–1.97
Median pedestrian route directness	1.28 (0.24)	1–2.32	1.28 (0.20)	1–1.91	1.29 (0.32)	1–4.56
Intersection density	85.76 (40.28)	10.34–200.26	77.05 (34.69)	9.46–152.69	71.46 (31.83)	14.44–137.16
Cul de sacs (count)	2.53 (2.21)	0–10.00	8.47 (5.57)	0–27.00	31.46 (16.45)	2–68.00
Average speed limit (mph)	27.70 (2.26)	23.93–34.69	28.04 (2.12)	22.35–35.26	28.43 (1.74)	23.27–35.37
Highway density	62.01 (112.50)	0–574.98	74.61 (90.42)	0–389.78	89.33 (73.31)	0–400.15
Residential density	117.60 (89.66)	5.71–373.94	113.88 (82.88)	5.98–285.51	104.41 (73.53)	6.75–258.73
Population density	2,666 (2,228)	82.46–8,346	2,617 (2,123)	87.15–7,162	2,386 (1,880)	97.85–6,020

Abbreviations: SD, Standard Deviation; M, Mean.

**Table 3:** Correlation between Walk Scores and GIS Neighborhood Walkability Indicators, including all data and for each geographic region.

	400-meter Network Buffer		800-meter Network Buffer		1600-meter Network Buffer	
	r_S_	p-value	r_S_	p-value	r_S_	p-value
**Overall (n = 733)**						
Retail destinations (density)	0.53	<0.0001	0.67	<0.0001	0.80	<0.0001
Service destinations (density)	0.27	<0.0001	0.53	<0.0001	0.67	<0.0001
Cultural/educational destinations (density)	0.44	<0.0001	0.53	<0.0001	0.69	<0.0001
Parks (density)	0.24	<0.0001	0.37	<0.0001	0.51	<0.0001
Median pedestrian route directness	0.24	<0.0001	−0.01	0.7908	−0.05	0.2166
Intersection density	0.51	<0.0001	0.59	<0.0001	0.65	<0.0001
Cul de sacs (count)	0.01	0.7024	0.14	0.0002	0.37	<0.0001
Average speed limit (mph)	0.47	<0.0001	0.53	<0.0001	0.47	<0.0001
Highway density	0.33	<0.0001	0.39	<0.0001	0.43	<0.0001
Residential density	0.65	<0.0001	0.65	<0.0001	0.65	<0.0001
Population density	0.64	<0.0001	0.64	<0.0001	0.64	<0.0001
**Pacific Northwest (n = 172)**						
Retail destinations (density)	0.45	<0.0001	0.64	<0.0001	0.78	<0.0001
Service destinations (density)	0.33	<0.0001	0.60	<0.0001	0.78	<0.0001
Cultural/educational destinations (density)	0.42	<0.0001	0.53	<0.0001	0.70	<0.0001
Parks (density)	0.19	0.0146	0.27	0.0003	0.38	<0.0001
Median pedestrian route directness	0.09	0.4232	−0.02	0.8426	−0.11	0.1496
Intersection density	0.29	<0.0001	0.42	<0.0001	0.49	<0.0001
Cul de sacs (count)	−0.09	0.2264	−0.02	0.7494	0.24	0.0014
Average speed limit (mph)	0.34	<0.0001	0.37	<0.0001	0.36	<0.0001
Highway density	0.23	0.0027	0.19	0.0116	0.32	<0.0001
Residential density	0.52	<0.0001	0.51	<0.0001	0.50	<0.0001
Population density	0.43	<0.0001	0.43	<0.0001	0.43	<0.0001
**Midwest (n = 167)**						
Retail destinations (density)	0.32	<0.0001	0.49	<0.0001	0.85	<0.0001
Service destinations (density)	0.34	<0.0001	0.53	<0.0001	0.69	<0.0001
Cultural/educational destinations (density)	0.27	0.0004	0.40	<0.0001	0.73	<0.0001
Parks (density)	−0.16	0.0382	−0.09	0.2302	0.14	0.0638
Median pedestrian route directness	0.19	0.1369	0.05	0.6009	0.17	0.0330
Intersection density	0.29	0.0002	0.12	0.1320	0.28	0.0002
Cul de sacs (count)	−0.13	0.0942	−0.03	0.6556	0.12	0.1115
Average speed limit (mph)	0.45	<0.0001	0.53	<0.0001	0.58	<0.0001
Highway density	0.18	0.0201	0.32	<0.0001	0.46	<0.0001
Residential density	0.74	<0.0001	0.73	<0.0001	0.71	<0.0001
Population density	0.70	<0.0001	0.68	<0.0001	0.67	<0.0001
**South (n = 230)**						
Retail destinations (density)	0.33	<0.0001	0.58	<0.0001	0.70	<0.0001
Service destinations (density)	0.25	0.0002	0.46	<0.0001	0.57	<0.0001
Cultural/educational destinations (density)	0.25	0.0002	0.29	<0.0001	0.49	<0.0001
Parks (density)	0.13	0.0531	0.26	<0.0001	0.35	<0.0001
Median pedestrian route directness	0.24	0.0185	0.08	0.2724	0.15	0.0259
Intersection density	0.17	0.0088	0.32	<0.0001	0.40	<0.0001
Cul de sacs (count)	−0.09	0.1979	−0.08	0.2228	0.10	0.1258
Average speed limit (mph)	0.26	<0.0001	0.34	<0.0001	0.28	<0.0001
Highway density	0.13	0.0494	0.13	0.0425	0.13	0.0467
Residential density	0.43	<0.0001	0.41	<0.0001	0.42	<0.0001
Population density	0.36	<0.0001	0.35	<0.0001	0.33	<0.0001
**East (n = 164)**						
Retail destinations (density)	0.56	<0.0001	0.70	<0.0001	0.73	<0.0001
Service destinations (density)	0.28	0.0003	0.47	<0.0001	0.56	<0.0001
Cultural/educational destinations (density)	0.60	<0.0001	0.74	<0.0001	0.83	<0.0001
Parks (density)	0.25	0.0010	0.41	<0.0001	0.69	<0.0001
Median pedestrian route directness	0.24	0.0099	0.15	0.0820	−0.09	0.2840
Intersection density	0.75	<0.0001	0.78	<0.0001	0.79	<0.0001
Cul de sacs (count)	0.09	0.2681	0.32	<0.0001	0.71	<0.0001
Average speed limit (mph)	0.41	<0.0001	0.39	<0.0001	0.33	<0.0001
Highway density	0.34	<0.0001	0.36	<0.0001	0.28	0.0003
Residential density	0.77	<0.0001	0.79	<0.0001	0.80	<0.0001
Population density	0.75	<0.0001	0.75	<0.0001	0.76	<0.0001
